# Targeting Tumors with Salmonella *Typhimurium* - Potential for Therapy

**DOI:** 10.18632/oncotarget.206

**Published:** 2011-01-03

**Authors:** Daniel M. Wall, C.V. Srikanth, Beth A. McCormick

**Affiliations:** ^1^Institute of Infection, Immunity and Inflammation, College of Medical, Veterinary and Life Sciences, University of Glasgow, G12 8QQ, United Kingdom; ^2^Department of Microbiology and Physiological Systems, University of Massachusetts Medical School, 55 Lake Avenue North, Worcester, MA 01655, United States of America

**Keywords:** Salmonella, cancer, therapy, tumor targeting, bacteria

## Abstract

When one considers the organism *Salmonella enterica* serotype Typhimurium (*S. Typhimurium*), one usually thinks of the Gram-negative enteric pathogen that causes the severe food borne illness, gastroentertitis. In this context, the idea of *Salmonella* being exploited as a cancer therapeutic seems pretty remote. However, there has been an escalating interest in the development of tumor-therapeutic bacteria for use in the treatment of a variety of cancers. This strategy takes advantage of the remarkable ability of certain bacteria to preferentially replicate and accumulate within tumors. In the case of *S. Typhimurium*, this organism infects and selectively grows within implanted tumors, achieving tumor/normal tissue ratios of approximately 1,000:1. *Salmonella* also has some attractive properties well suited for the design of a chemotherapeutic agent. In particular, this pathogen can easily be manipulated to carry foreign genes, and since this species is a facultative anaerobe, it is able to survival in both oxygenated and hypoxic conditions, implying this organism could colonize both small metastatic lesions as well as larger tumors. These observations are the impetus to a burgeoning field focused on the development of *Salmonella* as a clinically useful anti-cancer agent. We will discuss three cutting edge technologies employing *Salmonella* to target tumors.

## INTRODUCTION

Bacteria have been investigated as therapeutic agents for tumors for over 150 years, when it was first observed by William B. Coley that a fraction of cancer patients who developed post-operative bacterial infections went into remission and were cured of their tumors [1]. Although the mechanisms underlying this observation were unclear, it was known even then that bacteria exhibit immunostimulatory properties. Moreover, it has been known for nearly 50 years that anaerobic bacteria can selectively grow in tumors, underscoring the fact that such microbes have the potential to overcome many of the delivery barriers that hinder conventional chemotherapeutics [2]. In particular, the conditions that permit anaerobic bacterial growth, such as impaired circulation, and extensive necrosis, are found in many tumors signifying that bacterial therapeutic conduits may serve as a unique portal to a wide variety of malignancies. Recent advances in molecular biology, as well as the complete sequencing of many bacterial genomes, have fueled a resurgence of interest in bacteria as drug delivery vehicles and tumoricidal agents. Here we will highlight recent advances exploiting the use of the food-borne pathogen *Salmonella enterica* serotype Typhimurium (*S.* Typhimurium), as an intriguing chemotherapeutic agent.

## THE BENEFITS OF BACTERIAL CANCER THERAPY

Current chemotherapeutics (and radiation) used in the treatment of cancer patients have considerable limitations, including toxicity, poor tumor targeting, and inadequate tissue penetration, which together often result in incomplete destruction of the tumors. Selected bacteria, however, can be manipulated to overcome many of the limitations that frequently hamper current cancer regiments by directly targeting cancer cells, killing these cells through innate bacterial toxicity, competition for nutrients or delivery of anti-cancer agents. Salmonella, Clostridium, Escherichia, Bifodobacterium, Caulobacter and Listeria species have all been tested for their potential use in cancer therapy with varying degrees of success [3,4,5,6,7,8].

**Figure 1:**
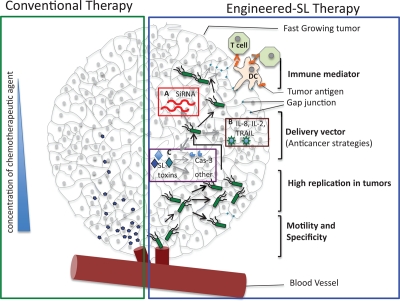
Schematic representing the advantages offered by *Salmonella* (SL) as a cancer therapeutic agent Green box represents how the conventional chemotherapeutic agent (blue pentagon) is unable to penetrate deep into the fast growing tumor. Blue box represents the engineered-Salmonella therapy. Salmonella offers several advantages like (i) tumor-specificity, (ii)self-replicating potential, (iii) ability to be genetically engineered with either (a) gene silencing (red box), or (b) tumor sensitizing (brown box) or (c) bacterial toxin/effectors producing methodologies (purple box), leading to tumor regression by caspase-3 mediated and other mechanisms, (iv) ability to migrate to distal regions of the tumor and (v) most importantly to induce gap-junction in tumors leading to immune activation against the tumor.

Bacterial toxins that are highly immunogenic have also been manipulated by linking them to tumor antigens in an attempt to elicit an immune response to tumors and sensitize the immune system to tumor presence [9,10]. Furthermore, bacteria such as *S.* Typhimurium have an innate advantage as a chemotherapeutic considering they are attracted to cancer cells by compounds released by necrotic or quiescent cells within the tumor, thereby they can be used to either infect cells or deliver compounds directly to the tumor site [8,11,12,13]. Interestingly, bacteria have also evolved to overcome several of the limitations of conventional therapy given that these organisms can penetrate deep into tumors and are unaffected by the immune evasion strategies deployed by tumors that can make cancer treatment so challenging (Figure 1).

## SALMONELLA TYPHIMURIUM AS A TREATMENT OPTION

Clostridium and Salmonella species have historically been used most successfully as anti-cancer microbes [1,2,14,15,16]. These organisms have been used directly as therapeutics, as well as delivery vehicles for anti-cancer therapeutics. However, recent advances in tumor targeting with attenuated *S.* Typhimurium have selected this pathogen as the bacterium of choice for cancer therapy development resulting in it being employed in human trials [17,18,19]. Moreover, unlike the strictly anaerobic Clostridium which must be delivered in spore form, *S.* Typhimurium is motile, easily genetically manipulated, and grows as a facultative anaerobe in the presence or absence of oxygen. These and other advantages (summarized in Table 1) allow *S.* Typhimurium to not only target tumors, but also deliver therapeutic compounds or immune modulators to the tumor site.

**Table 1:** Advantages of Salmonella as an anti-tumor agent

Feature	Underlying Reason	References
Systemic administration	VNP20009 can be delivered intravenously or by direct injection into a tumor	[17,35,44]
Tumor specificity	Salmonella can accumulate at levels 1,000 fold higher in tumors as opposed to normal tissues reducing the risk of toxic side effects of proteins or compounds delivered systemically.	[21,45,46]
Replication competent	Unlike other therapeutic agents Salmonella replicates at the tumor site so that a low? dose replicates to an effective dose within the target tumor	[24,44,45,47]
Broad tumor specificity	Salmonella targets a broad range of solid tumors, including melanoma, lung, colon, breast, renal, hepatic, and prostate tumors	[22,48,49,50,51,52,53,54,55]
Delivery capacity	Salmonella is metabolically active and can continuously produce a protein of interest during infection of the tumor	[10,37,56,57,58]
Anti-tumor Immune activation	Salmonella infection induces the upregulation of Cx43 protein. This results in the formation of functional Gap?junctions in tumors leading to transfer of tumor antigenic peptides to the DCs and eventually recruiting CD8 T cells	[32,34,59]
Antibiotic sensitivity	Salmonella can be easily removed following treatment with antibiotics Native cytotoxicity Ability of the bacteria to produce virulence factors that leads to cytotoxicity and attract immune cells to the tumors helps in further tumor regression	[50,60]
Post delivery detection	Engineered Salmonella expresses fluorescent proteins that offers the ability to be externally detected	[47,61,62,63]

The anoxic environment often found in tumors makes the tumor site an attractive niche for *S.* Typhimurium growth. Through the use of their flagella, *Salmonella* migrate towards the tumor, attracted by the high concentrations of nutrients available within the tumor microenvironment [20,21,22]. The metabolism of *S.* Typhimurium has been exploited to attenuate the strains used in cancer therapy, manipulating the bacteria so that they grow only at tumor sites. Metabolic genes have been removed from *S.* Typhimurium, such as *purI* in the mutant strain VNP20009, rendering the mutant bacterium auxotrophic for certain compounds that are found in very high concentrations at tumor sites, in this case purines [23]. Such genetic engineering, forces migration of *S.* Typhimurium towards tumors in order to survive and results in the bacteria accumulating at the tumor site at over a 1,000 times higher levels than in normal tissue [24].

Migration towards the tumor site is based on the ability of *S.* Typhimurium to sense nutrients using a number of receptors that are located at the bacterial poles on the outer membrane of the bacteria. Two notable receptors have been characterized; the TAR receptor, which detects aspartate secreted by cancerous tissues and the TRG receptor, which aids in migration towards ribose found in necrotic tissues [21]. Further manipulation of this ability to chemotax towards tumors may lead to the development of bacteria that are directed toward specific regions within tumors. Towards this end, it was discovered that the aspartate receptor controls migration towards tumors, the serine receptor initiates penetration, and the ribose/galactose receptor directs *Salmonella* into necrotic regions [21]. Therefore knocking out a particular receptor may help direct *Salmonella* to particular region within the tumor. Other alterations, such as truncation of lipid A [23], reduces the immunogenicity of the bacteria, which in turn, reduces the risk of an adverse inflammatory reaction and possible toxic shock.

## EXPLOITING SALMONELLA TYPHIMURIUM TUMOR TARGETING

*S.* Typhimurium, once at the tumor site, is thought to help destroy tumors through competition for nutrients and innate toxicity as a result of damage due to growth of the bacteria and production of toxins. Presently, *S.* Typhimurium is being used to target and destroy tumors in three specific ways: i) delivery of anti cancer compounds, ii) sensitizing the immune system to the presence of tumors, and iii) using bacterial toxins to directly activate caspase-3, a key enzyme of the apoptotic pathway (Figure 1).

### Using Salmonella to deliver anti cancer compounds

Bacteria have been recently described as “tiny programmable robot factories” for use in cancer therapy [25]. Indeed, there are numerous compounds that can be delivered via bacteria to a tumor site including cytotoxic agents, green fluorescent protein for targeting and visualization of tumors, DNA for gene therapy, and small interfering RNAs (siRNA) to target expression of key proteins within tumors [11,22,26,27,28,29,30,31]. While these techniques have had varying levels of success in animal models, it is inferred that these practices when used in combination with the native toxicity of *Salmonella* may offer a fascinating alternative to traditional gene therapy approach.

It is envisaged that specific anti-cancer molecules delivered by *S*. Typhimurium can be used to target the outer regions of the tumor with the bacteria invading deep within the tumor, penetrating regions of the tumor architecture that conventional therapies cannot reach [12,32]. One of the key challenges with conventional chemotherapy is delivery of potentially toxic agents to the appropriate areas of the tumor while preventing damage to healthy tissue. Temporal control over bacterial delivery of therapeutic agents is a key consideration as delivery of these compounds during transit to the tumor site will distribute products systemically and increase toxicity in healthy tissues. Inducible gene promoters offer a solution that is presently employed. Detection of small molecules by the bacteria, or irradiation at the point where expression is required, have both been used successfully *in vivo* along with the use of pro-drugs introduced at the tumor site for conversion into an anticancer agent by the bacteria upon contact [13,32,33]. *recA,* the radiation inducible promoter, was linked to tumor necrosis factor (TNF)-related apoptosis inducing ligand (TRAIL) to control its expression by *Salmonella* during infection of tumors [32]. *S*. Typhimurium at the tumor site was then induced to express TRAIL by doses of radiation, which simultaneously treated the tumor. *S.* Typhimurium promoters specifically induced by hypoxia have been identified and may be of use in the future, with bacterial expression of a therapeutic compound activated once the bacteria reach the tumor [33].

A second approach for controlling therapeutic delivery is the construction of bacteria that produce enzymes converting harmless pro-drugs into active agents inside the tumor. Toward this goal *Salmonella* has been engineered to express cytosine deaminase (CDase) that cleaves the pro-drug 5-fluorocytosine to the active chemotherapeutic 5-fluorouricil [13,18].

### Using Salmonella to sensitize the immune system to the presence of tumors

The ignorance of the immune system to the presence of tumors within the body is a significant consideration in attempting to treat cancer. While numerous methods have been attempted to alert the immune system to the presence of tumors, recent work has shed new light on the role that *Salmonella*, in particular, plays in this process. *S*. Typhimurium infection of cancer cells was shown to upregulate the cellular protein connexin43, resulting in gap junction formation not only between tumor cells but also between cancer cells and antigen presenting cells (APCs) [34]. This allows APCs access to pre-processed tumor antigens, which they can then present to T-cells, sensitizing the immune system to the presence of tumors and activating an anti-tumor response. This ability to sensitize the immune system to the presence of tumors may explain some of the success *Salmonella* has in combating tumors. Alternative methods of sensitizing the immune system through *Salmonella* targeting of tumors have also been used.

Other approaches employ *S.* Typhimurium to deliver cytokines to the tumor site in an attempt to activate immune cells or elicit an immune response against the tumor [8,10,35,36,37,38,39]. Antigens have also been linked to bacterial toxins that are highly immunogenic to sensitize the immune system against cancer cell antigens [10,40]. Again, this approach harnesses the ability of *S*. Typhimurium to deliver immune sensitizing compounds at the tumor site to alert circulating immune cells, while at the same time the bacteria target the more difficult to reach anoxic regions of the tumor.

### Using bacterial toxins to directly activate caspase-3

It is becoming increasingly evident that the ability to control or inhibit apoptosis affords a distinct advantage to certain bacterial pathogens, as it allows the organism to replicate within the infected cell, increasing the chance of a successful infection. Although a number of bacteria specifically inhibit apoptosis, our recent evidence indicates that *S.* Typhimurium may activate specific apoptotic enzymes [41]. We found that activation of caspase-3 by a single effector protein of *S.* Typhimurium increases the infectivity of this pathogen, as caspase-3 directed the processing of *S.* Typhimurium secreted effectors into their functional subunits upon their delivery into the host cell [42]. Such an unprecedented means of promoting infection indicates that the interaction of bacteria with apoptotic pathways may be more intimate than previously recognized, and in fact, may be quite common amongst pathogens.

This observation raises a key question with regards to *S*. Typhimurium therapy for cancer - can we use bacteria to exploit apoptotic pathways, and in particular activation of caspase-3, opening a new front in the fight against cancer? Direct activation of caspase-3 in the treatment of cancer through using procaspase-3 activating compounds has been attempted previously using pan activating caspase-1 (PAC-1) [43]. Thus, employing an efficient bacterial vector to directly deliver a native bacterial effector for caspase-3 activation offers a different approach to bacterial therapy. Rather than deliver large quantities of non native proteins, it may be possible to channel the built-in toxicity of *S.* Typhimurium to destroy tumors. *S.* Typhimurium also harbors several promoters that are induced under hypoxic conditions, and using these promoters to control expression of specific effectors may offer novel opportunities to utilize the innate bacterial toxicity. In particular, *S.* Typhimurium may perhaps be engineered to up-regulate caspase-3 through effector expression while at the same time introducing potent anti-cancer drugs that can no longer be successfully expelled by the tumor.

## FUTURE DIRECTIONS FOR SALMONELLA CANCER THERAPY

In sum, bacterial cancer therapy has made great strides in past decade, and is now considered a tangible option for future cancer therapy. The potential for bacterial therapy seems endless but some fundamental issues need to be reconciled before this kind of therapy moves into a clinic setting. Insufficient colonization of tumors appears to be the major obstacle identified in clinical trials, a problem that was not evident in animal models. Overcoming this challenge is one of the priorities in developing bacteria as cancer therapy agents. But the ease of genetic manipulation in *S.* Typhimurium may prove to be the key in surmounting this hurdle. Toxicity of *S.* Typhimurium is also problematic, with the bacteria too attenuated to destroy tumors once at the tumor site due to toxin removal, or poor expression of anti-cancer compounds, or the risks of toxicity are too great for injection of *S.* Typhimurium into severely immunocompromised patients. Overcoming these limitations, particularly with respect to using bacterial proteins in therapy, are key to moving this aspect of bacterial cancer therapy forward.
